# On the Hydrated Peroxide of Iron and Magnesia as Antidotes in Poisoning with Arsenic

**Published:** 1852-08

**Authors:** J. Haidlen


*On the Hydrated Peroxide of Iron and Magnesia as Antidotes in Poisoning with Arsenic.* By J. Haidlen.—The author has satisfied himself by experiments, that to completely precipitate 1 part of arsenious acid within five minutes by hydrated oxide of iron, 22 parts of the latter are required. Some of which had been kept for a year, had lost more than two-thirds of its power; in another case it had lost one-third of it. As is well known, on account of this property of the hydrated oxide of iron, Fuchs has recommended the mixture of hydrated peroxide of iron and magnesia, *i. e.* the mixture of a solution of persulphate of iron, which yields from 17 to 18 per cent. of the hydrated peroxide dried at 212° F., with excess of caustic magnesia. In the Wurtemberg Pharmacopœia the mixture of perchloride of iron with excess of of magnesia is prescribed. The author finds that both mixtures precipitate the arsenious acid equally as well as the pure hydrated peroxide of iron, so that there is no chemical objection to their use.—*Jahrb. für Prakt. Pharm.*, vol. xxiii. pp. 196–202.

